# Epilepsy Cured by Extracting a Foreign Body from the Meatus Auditorious

**Published:** 1845-03

**Authors:** 


*Epilepsy cured by extracting a foreign body from the Meatus Auditorious* — The following curious case reported in a Bordeaux Medical Journal, by Dr. Roussilhe, will be read with interest: Jean Raynes, a farmer, aged thirty years, introduced a small stone or pebble into his left ear in 1833. For a long time he was only troubled with partial deafness; by-and-by the auditory canal became the seat of a discharge to which the patient paid little or no attention. In 1836 he was seized with vertigo, which forced him to suspend his occupation. About the period of the harvest, he was assailed by a violent attack of *epilepsy,* since which time these attacks have come on with the greatest irregularity. In 1838, Dr. Roussilhe was consulted. The epileptic seizure was always preceded by symptoms of vertigo. The mother of the patient being present during the consultation, alluded to the introduction of the foreign body into the ear some time before the epileptic attack. Recollecting the case related by *Fabricius de Hildanus,* in which the extraction of a piece of glass from the ear of a young girl had cured an attack of epilepsy, Dr. R. proposed to extract it in this case. On examining the ear with a speculum, he found a large mass of cerumen lodged in the bottom of the auditory canal. The canal was slightly inflamed, and also the seat of a sero-purulent discharge. An alcholic solution of soap was thrown into the ear with a view to dissolve and remove this indurated cerumen. With the aid of a blunt pointed stylet, the ear was thoroughly cleansed. After this, the foreign body was soon reached, and the operator endeavored, but in vain, to pass the stylet beyond its great diameter, and to extract it; he could only move it from its bed. He allowed the patient to rest for a few moments, because he had suffered a good deal during these efforts. He then filled the auditory canal with warm olive oil, and allowed it to remain there for some moments. He took a small silver wire and bent it in the middle, so as to form a sort of noose, and then introduced it down to the foreign body, and attempted to pass it beyond it; this was soon and easily effected; he next drew gently upon the noose, and secured the body and withdrew it at once. It proved to be a small stone or pebble of irregular shape and nearly a triangular form. This body had remained *five years*

in the ear. He advised the patient to throw emollient injections into the auditory canal, and to take daily the infusion of valerian. Since that period up to the time the case was recorded, (September, 1843,) the epilepsy had not re-appeared. M. Roussilhe is induced to believe the disease will not return.—*Bulletin Therapeutique.—New Orleans Med. Jour.*

